# Efficacy, safety and tolerability of drugs studied in phase 3 randomized controlled trials in solid tumors over the last decade

**DOI:** 10.1038/s41598-021-90403-3

**Published:** 2021-05-25

**Authors:** Domen Ribnikar, Hadar Goldvaser, Zachary W. Veitch, Alberto Ocana, Arnoud J. Templeton, Boštjan Šeruga, Eitan Amir

**Affiliations:** 1 Department of Medical Oncology, Institute of Oncology Ljubljana, Ljubljana, Slovenia; 2 Division of Medical Oncology and Hematology, University of Toronto and Princess Margaret Cancer Centre, 610 University Ave, 700U, 7-721, Toronto, ON M5G 2M9 Canada; 3 Rabin Medical Center, Davidoff Cancer Center, Beilinson Hospital, Petah Tikva, Israel; 4 Sackler Faculty of Medicine, Tel Aviv University, Tel Aviv, Israel; 5 Drug Development Program, Hospital Clinico San Carlos and CIBERONC, Madrid, Spain; 6 Translational Oncology Laboratory. Regional Center for Biomedical Research (CRIB), Castilla La Mancha University, Albacete, Spain; 7 Department of Oncology, St. Claraspital Basel, Basel, Switzerland; 8 Faculty of Medicine, University of Basel, Basel, Switzerland

**Keywords:** Cancer, Oncology

## Abstract

Data suggest that for newly approved cancer drugs safety and tolerability are worse than in control arms of registration trials. Less is known about the balance between efficacy and toxicity of drugs studied in unselected phase 3 randomized controlled trials (RCTs) including those not resulting in regulatory approval. We searched Clinicaltrials.gov to identify phase 3 RCTs in patients with advanced breast, colorectal, lung, or prostate cancer completed between January 2005 and October 2016. We extracted efficacy and safety data from publications. For efficacy hazard ratios (HRs) for progression-free survival (PFS) and overall survival (OS) were extracted. For safety, we computed odds ratios (ORs) and 95% confidence intervals (CIs) for toxic death, treatment discontinuation without progression and commonly reported grade 3/4 adverse events (AEs). Data were then pooled in a meta-analysis. Of 377 RCTs identified initially, 143 RCTs comprising 88,603 patients were included in the analysis. Of these, 79 (57%) trials met their primary endpoint. Compared to control groups, both PFS (HR 0.80; 95% CI 0.78–0.82) and OS (HR 0.87; 95% CI 0.85–0.89) were improved with experimental drugs. Toxic death (OR 1.14; 95% CI 1.03–1.27), treatment discontinuation without progression (OR 1.64; 95% CI 1.56–1.71) and grade 3/4 AEs were also more common with experimental drugs compared to respective control group therapy. Just over half of phase 3 RCTs in common solid tumors met their primary endpoint and in nearly half, experimental therapy had worse safety compared to control arms.

## Introduction

Despite improvements in outcome, in most cases, metastatic cancer remains an incurable disease. While efficacy of experimental therapy remains of primary interest, a focus on toxicity of cancer drugs which could adversely influence quality of life and even increase non-cancer mortality is warranted in the setting of incurable cancer. Randomized controlled trials (RCTs) are the recognized gold standard to evaluate the efficacy and safety of new treatments^1,2^. Often, toxicity data from RCTs are insufficient and can be misleading^3^. Large RCTs are not designed to detect statistically significant differences in toxicity between standard and experimental arms. Furthermore, rare but potentially life-threatening adverse events may not be identified in RCTs^4^.

It has been demonstrated that newly approved anticancer drugs increase morbidity and treatment-related mortality^5^, however there are few data about toxicity of experimental cancer drugs in unselected trials including those not resulting in drug registration. Here, we report a meta-analysis of efficacy, safety and tolerability of all phase 3 RCTs in common advanced solid tumors registered on ClinicalTrials.gov. The primary objective of the study was to quantify systematically the trade-off between efficacy and toxicity of experimental cancer therapy relative to control group treatment in unselected phase 3 trials. We hypothesized that a small incremental benefit of experimental cancer drugs would be associated with higher toxicity.

## Methods

### Search strategy

We searched Clinicaltrials.gov^6^ to identify phase 3 RCTs evaluating new drugs in adult patients with advanced breast, colorectal, lung, or prostate cancer. We included trials categorized as completed or active with accrual completed between January 1, 2005 and October 31, 2016. Consistent with prior methodology^7^, studies evaluating supportive care agents, studies with different scheduling and/or dosing of the same agent, single arm studies, trials not evaluating systemic therapy (such as trials exploring radiation, surgery, imaging [including screening] and chemoprevention) or those consisting exclusively of biomarker, pharmacokinetic and pharmacodynamics analyses were excluded. Trials listed as active but not recruiting and without available results were excluded as well.

### Data extraction and synthesis

We utilized methods similar to those used previously in analyses for approved drugs^5^. Briefly, full text articles of eligible studies from the literature were retrieved and the primary data for efficacy, safety and tolerability were extracted independently by two coauthors, DR and HG. Disagreement was resolved by consensus. For efficacy endpoints, we extracted hazard ratios (HRs) and corresponding 95% confidence intervals (CI) for progression-free survival (PFS) and/or overall survival (OS). We extracted data only for the primary endpoint of each study. For safety and tolerability, first we identified the number of patients at risk both, in the experimental and control arms, and then collected data on the number of patients with each of the following safety and tolerability outcomes: treatment-related death, treatment discontinuation without disease progression, and 12 commonly reported grade 3/4 adverse events (AEs) including: anemia, neutropenia, thrombocytopenia, diarrhea, vomiting, stomatitis, hypertension, cardiac events, fatigue/asthenia, skin toxicity, dyspnea, neuropathy. Subsequently, we calculated the odds ratios (ORs) comparing the experimental and control groups for each safety and tolerability outcome measure.

Assessment of study quality or risk of bias was not performed routinely as all included studies were large randomized trials and those which were open-label studies were often appropriately unblinded (e.g. substantial differences in toxicity profile between experimental and control drugs making blinding ineffective). In sensitivity analyses, the impact on concealment and the potential for the placebo effect were explored (see below).

### Statistical analysis

Data were presented descriptively as absolute numbers, proportions, and ranges, as appropriate. Data were pooled in a meta-analysis using RevMan version 5.3 (Cochrane Collaboration, Copenhagen, Denmark). For efficacy analyses, pooled estimates of HRs were computed and weighted using generic inverse variance approach^8^ and random-effect modelling^9^. Analyses of safety and tolerability were computed using different methods for toxic death, treatment discontinuation and grade 3/4 AEs. For toxic death where absolute event rates were less than 1%, the Peto one-step odds ratio method was utilized^8,10^. For treatment discontinuation where there were low absolute event rates and substantial variability in relative effect-sizes, the Mantel–Haenszel odds ratio method was used^9^. Finally, for grade 3/4 AEs, the DerSimonian and Laird random-effects method was utilized and studies were weighted using the generic inverse variance approach^8^. Sensitivity analyses excluding open-label studies and those which were not placebo-controlled were performed. We also performed additional subgroup analyses of efficacy and safety outcomes based on cancer site. Associations between efficacy and toxicity were assessed using meta-regression which comprised a univariable linear regression of the natural logarithm of the HR for efficacy endpoints and the natural logarithm of the OR for toxicity outcomes. Regression was weighted by individual study sample size using the weighted least square (mixed effect) function^11^. Statistical analyses were conducted using SPSS statistical software, version 21 (IBM Corp, Armonk, NY). All statistical tests were two-sided, and statistical significance was defined as p < 0.05. No corrections were made for multiple statistical testing.

### Conference presentation

This study was presented in part at the 2018 American Society of Clinical Oncology Annual Meeting (Ribnikar et al. J Clin Oncol 2018; 35(15_Suppl); Abstract 6588.

## Results

**Figure 1:**
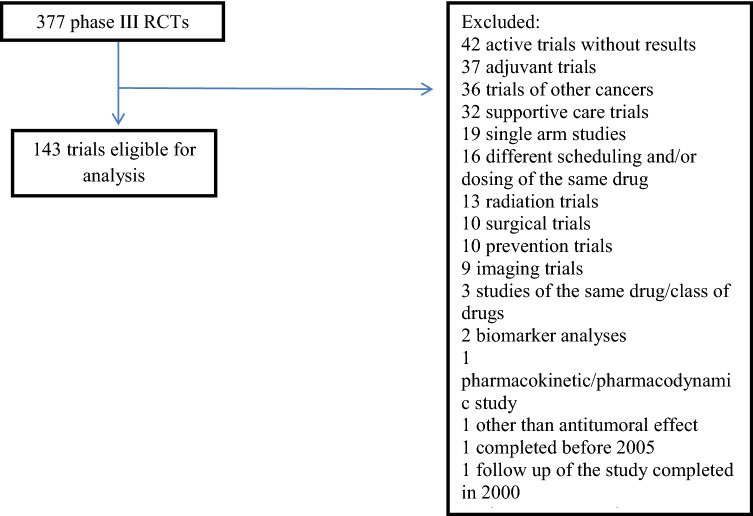
Study selection.

A total of 377 RCTs were identified initially. After excluding ineligible studies, a total of 143 studies comprising 88,603 patients were included in the analysis (see Fig. 1 for study selection schema and PRISMA flow diagram). The characteristics of included trials are presented in Table 1. The details of the 143 trials that were included in the analysis are presented in the Supplementary Table 1. Among the 377 trials identified in clinicaltrials.gov published results could not be identified for 42 studies (11%). This likely reflects publication bias.

**Table 1:** Characteristics of included trials.

Variable	All studies (n = 143)	Chemotherapy studies (n = 30)	Endocrine studies (n = 12)	Studies with targeted agents (n = 85)	Immunotherapy studies (n = 9)	Studies evaluating other agents (n = 7)
N of pts	88,603	17,676	8017	53,825	5748	3337
**Primary endpoint**						
PFS	68	11	3	52	1	1
OS	64	17	5	30	8	4
**Cancer site**						
Breast	39 (27%)	8 (27%)	3 (25%)	28 (33%)	0 (0%)	0 (0%)
Colorectal	21 (15%)	3 (10%)	0 (0%)	17 (20%)	0 (0%)	1 (14%)
Lung	57 (40%)	16 (53%)	0 (0%)	35 (41%)	4 (44%)	2 (29%)
Prostate	26 (18%)	3 (10%)	9 (75%)	5 (6%)	5 (56%)	4 (57%)
**Trial outcome**						
Positive	79 (57%)	15 (50%)	8 (73%)	49 (59%)	4 (44%)	3 (43%)
Negative	60 (43%)	15 (50%)	3 (27%)	34 (41%)	5 (56%)	3 (43%)
**Concealm. method**						
Blinding	63 (44%)	3 (10%)	6 (50%)	43 (51%)	7 (78%)	4 (57%)
Open-label	80 (56%)	27 (90%)	6 (50%)	42 (49%)	2 (22%)	3 (43%)

### Efficacy

PFS was the primary endpoint in 68 of trials (48%) and data on PFS were reported in 60 studies (42%). PFS was significantly improved with experimental therapy in 35 (58%) studies. Overall, experimental drugs were associated with a 20% relative improvement in PFS in comparison to control drugs (HR 0.80; 95% CI 0.78–0.82). Sensitivity analysis excluding open-label studies (i.e. only including blinded trials) did not change results substantially (HR 0.75; 95% CI 0.72–0.78). A sensitivity analysis excluding placebo-controlled trials showed that experimental drugs were associated with a 14% relative improvement in PFS in comparison to control drugs (HR 0.86; 95% CI 0.83–0.89).

OS was the primary endpoint in 64 trials (45%) and data on OS were reported in 56 (39%) studies. OS was significantly improved with experimental therapy in 26 (46%) studies resulting in a 13% relative improvement in overall survival (OS) compared to control agents (HR 0.87; 95% CI 0.85–0.89). Sensitivity analysis excluding open-label studies showed similar results (HR 0.87; 95% CI 0.85–0.90) as did sensitivity analysis excluding placebo-controlled trials (HR 0.88; 95% CI 0.85–0.92).

A subgroup analysis of efficacy outcomes according to cancer site demonstrated a similar trend as the overall analysis with improved PFS and OS in all cancer sites except in prostate cancer where we observed a worse PFS in experimental group (HR 1.45; 95% CI 1.00–2.11) in comparison to control group (see Table 2 for details regarding efficacy and safety outcomes according to cancer site).

**Table 2:** Hazard ratios (HRs) and odds ratios (ORs) and their 95% confidence intervals (CIs) for efficacy and safety outcomes based on the site of cancers.

Outcome	HR (for PFS, OS) OR (for toxicity)	95%CI
**(a) Breast cancer**		
PFS	0.82	0.79–0.85
OS	0.73	0.65–0.81
Toxic death	0.77	0.56–1.07
Treatment discontinuation	1.85	1.68–2.04
Anemia	1.47	1.09–1.98
Neutropenia	2.63	1.52–4.52
Thrombocytopenia	4.13	2.28–7.48
Diarrhea	1.28	0.84–1.95
Vomiting	1.30	0.95–1.76
Stomatitis	1.67	0.87–3.24
Hypertension	2.39	1.24–4.62
Cardiac events	1.44	0.95–2.20
Fatigue/asthenia	1.48	1.09–2.02
Skin toxicity	1.11	0.75–1.65
Dyspnea	0.99	0.72–1.37
Neuropathy	2.25	0.80–6.35
**(b) Prostate cancer**		
PFS	1.45	1.00–2.11
OS	0.83	0.80–0.87
Toxic death	1.20	0.95–1.51
Treatment discontinuation	1.60	1.47–1.73
Anemia	1.22	0.96–1.53
Neutropenia	1.41	0.68–2.90
Thrombocytopenia	1.49	0.71–3.12
Diarrhea	1.96	0.98–3.91
Vomiting	1.11	0.63–1.96
Stomatitis	13.60	6.71–27.58
Hypertension	2.83	1.95–4.11
Cardiac events	1.49	0.85–2.64
Fatigue/asthenia	1.14	0.89–1.47
Skin toxicity	5.67	1.81–17.78
Dyspnea	1.52	1.03–2.24
Neuropathy	0.74	0.35–1.55
**(c) Lung cancer**		
PFS	0.72	0.69–0.75
OS	0.94	0.91–0.97
Toxic death	1.13	0.97–1.32
Treatment discontinuation	1.54	1.42–1.66
Anemia	0.94	0.70–1.27
Neutropenia	0.60	0.43–0.82
Thrombocytopenia	1.43	0.89–2.29
Diarrhea	2.80	1.99–3.92
Vomiting	1.10	0.82–1.46
Stomatitis	2.63	1.32–5.25
Hypertension	2.55	1.36–4.78
Cardiac events	1.48	1.02–2.15
Fatigue/asthenia	1.30	1.11–1.53
Skin toxicity	5.26	3.07–9.02
Dyspnea	0.98	0.84–1.14
Neuropathy	0.78	0.34–1.82
**(d) Colorectal cancer**		
PFS	0.88	0.82–0.93
OS	0.81	0.76–0.87
Toxic death	1.46	1.07–2.00
Treatment discontinuation	2.03	1.79–2.31
Anemia	1.76	0.49–6.31
Neutropenia	0.94	0.60–1.48
Thrombocytopenia	3.06	0.93–10.03
Diarrhea	2.05	1.53–2.75
Vomiting	1.35	1.07–1.71
Stomatitis	2.69	1.68–4.31
Hypertension	2.78	1.30–5.93
Cardiac events	1.39	1.01–1.91
Fatigue/asthenia	1.31	0.95–1.80
Skin toxicity	10.25	3.85–27.28
Dyspnea	0.83	0.46–1.50
Neuropathy	0.48	0.22–1.02

### Toxicity

Data about individual grade 3/4 AEs were reported in all studies, however 9 (6%) studies did not report data on toxic deaths and 18 (13%) studies did not report data on treatment discontinuation. Overall, compared to control groups in individual studies, experimental drugs were associated with higher odds of toxic death, treatment discontinuation without progression, and most grade 3/4 AEs (see Table 3). A sensitivity analysis exploring the effect of blinding on toxicity data is shown in Table 4. There were no differences between blinded and open label studies for toxic death, however, blinded studies showed higher odds for treatment discontinuation and the following grade 3/4 adverse events: neutropenia, thrombocytopenia, diarrhea, stomatitis, hypertension, skin toxicity and neuropathy. A sensitivity analysis excluding placebo-controlled trials also showed higher odds for toxic death, treatment discontinuation and all grade 3/4 AEs (see Table 5), but odds were especially higher for thrombocytopenia, diarrhea, stomatitis, hypertension, cardiac events and skin toxicity.

**Table 3:** Odds ratios (ORs) and 95% confidence intervals (CIs) for safety and tolerability end points of experimental drugs in comparison to control groups.

Toxicity	OR	95% CI	p	Trials with significantly higher odds in the experimental arm	Trials with significantly lower odds in the experimental arm
Toxic death	1.14	1.03–1.27	0.02	5%	0%
Treatment discontinuation	1.64	1.56–1.71	< 0.001	46%	7%
Anemia	1.15	0.96–1.38	0.13	14%	11%
Neutropenia	1.09	0.86–1.39	0.47	28%	25%
Thrombocytopenia	2.04	1.46–2.85	< 0.001	16%	2%
Diarrhea	1.97	1.59–2.42	< 0.001	33%	5%
Vomiting	1.19	1.01–1.41	0.04	8%	3%
Stomatitis	2.44	1.69–3.51	< 0.001	25%	4%
Hypertension	2.63	1.93–3.60	< 0.001	42%	4%
Cardiac	1.46	1.19–1.78	< 0.001	9%	0%
Fatigue/asthenia	1.30	1.16–1.46	< 0.001	24%	5%
Skin	3.58	2.53–5.07	< 0.001	43%	8%
Dyspnea	1.04	0.91–1.19	0.52	6%	2%
Neuropathy	1.14	0.66–1.96	0.65	11%	11%

**Table 4:** Results of sensitivity analysis according to concealment method.

Toxicity	OR for blinded studies (n = 63)	95% CI for blinded studies	OR for open-label studies (n = 80)	95% CI for open-label studies
Toxic death	1.18	1.05–1.33	1.03	0.83–1.28
Treatment discontinuation	2.03	1.91–2.15	1.25	1.16–1.34
Anemia	1.14	0.92–1.41	1.14	0.85–1.51
Neutropenia	1.98	1.52–2.59	0.70	0.50–0.99
Thrombocytopenia	2.52	1.58–4.00	1.84	1.19–2.83
Diarrhea	2.63	1.95–3.55	1.54	1.15–2.06
Vomiting	1.22	0.99–1.50	1.13	0.89–1.44
Stomatitis	3.88	2.31–6.53	1.67	0.99–2.81
Hypertension	3.84	2.85–5.16	1.30	0.81–2.09
Cardiac	1.40	0.99–1.98	1.44	1.14–1.81
Fatigue/asthenia	1.41	1.21–1.64	1.19	1.00–1.41
Skin	4.31	2.73–6.80	3.07	1.89–5.00
Dyspnea	1.04	0.86–1.25	1.07	0.89–1.28
Neuropathy	1.58	0.92–2.71	0.82	0.36–1.84

**Table 5:** Results of sensitivity analysis based on placebo-control.

Toxicity	OR after excluding placebo-controlled trials (n = 80)	95% CI for excluded placebo-controlled trials	OR for the placebo-controlled trials (n = 80)	95% CI for placebo-controlled trials
Toxic death	1.12	0.91–1.38	1.15	1.02–1.28
Treatment discontinuation	1.28	1.20–1.37	2.05	1.93–2.18
Anemia	1.04	0.79–1.35	1.26	1.01–1.57
Neutropenia	0.73	0.53–1.01	2.26	1.66–3.07
Thrombocytopenia	1.84	1.19–2.83	2.52	1.58–4.00
Diarrhea	1.59	1.20–2.11	2.64	1.94–3.59
Vomiting	1.13	0.90–1.41	1.25	0.99–1.58
Stomatitis	1.78	1.06–3.00	3.68	2.17–6.24
Hypertension	1.46	0.88–2.43	3.81	2.83–5.14
Cardiac	1.67	1.33–2.10	1.56	1.12–2.16
Fatigue/asthenia	1.18	1.00–1.38	1.45	1.24–1.70
Skin	2.95	1.87–4.65	4.86	3.00–7.88
Dyspnea	1.02	0.86–1.20	1.08	0.88–1.34
Neuropathy	0.96	0.42–2.20	1.18	0.86–1.60

An additional subgroup analysis regarding safety outcomes according to the cancer site demonstrated higher odds for almost all toxicity outcomes with experimental drugs in all cancer sites, except for toxic death in breast cancer, neuropathy in prostate, lung and colorectal cancer and neutropenia in lung cancer. Odds were especially higher for thrombocytopenia in breast cancer, skin toxicity and stomatitis in prostate cancer, diarrhea and skin toxicity in lung cancer and thrombocytopenia, hypertension and skin toxicity in colorectal cancer (see Table 2 for details regarding odds for individual toxicity according to cancer site).

### Associations between efficacy and toxicity

We did not identify any statistically significant associations between PFS and any of the endpoints of toxicity. However, there was a statistically significant positive association between the HR for OS and the OR for treatment discontinuation without progression and for skin toxicity and a negative association with thrombocytopenia (see Table 6).

**Table 6:** Associations between efficacy and toxicity. β refers to the linear regression co-efficient.

Variable	β	P value
**Progression-free survival**		
Toxic death	0.083	0.60
Treatment discontinuation	− 0.041	0.79
Anemia	0.186	0.30
Neutropenia	− 0.038	0.81
Thrombocytopenia	0.453	0.078
Diarrhea	0.000	0.10
Vomiting	0.198	0.22
Stomatitis	0.072	0.73
Hypertension	− 0.329	0.15
Cardiac events	0.054	0.83
Asthenia/fatigue	− 0.120	0.45
Skin toxicity	− 0.021	0.90
Dyspnea	0.029	0.89
Neuropathy	− 0.281	0.33
**Overall survival**		
Toxic death	0.276	0.058
Treatment discontinuation	0.348	0.017
Anemia	− 0.093	0.55
Neutropenia	− 0.308	0.053
Thrombocytopenia	− 0.401	0.047
Diarrhea	0.209	0.16
Vomiting	0.141	0.44
Stomatitis	0.350	0.086
Hypertension	0.075	0.75
Cardiac events	0.174	0.44
Asthenia/fatigue	0.113	0.44
Skin toxicity	0.393	0.032
Dyspnea	0.258	0.22
Neuropathy	− 0.397	0.093

## Discussion

The main goal of phase 3 RCTs is to assess the efficacy of experimental therapy, however in the palliative treatment of patients with advanced cancer, where maintaining a good quality of life is crucial, toxicity profile and tolerability of drugs are of considerable importance. A modestly effective anticancer agent which adds significant toxicity and attenuates quality of life may not provide a favorable balance between benefits and risks.

In this study, we quantified the efficacy, safety and tolerability of experimental anti-cancer drugs evaluated in phase 3 RCTs in common solid tumors over almost 12 years. Results show that only 57% of phase 3 RCTs resulted in a significant improvement in their primary endpoint. While the estimate of around 50% success rate is consistent with prior published data^12^, given that phase 3 trials were likely supported by positive phase 1 and 2 data, we consider that a 57% success rate is disappointing. Pooled data show a 20% relative improvement in the hazards of progression and a 13% relative improvement in the hazards of death with experimental agents compared to controls. In contrast, we demonstrated that experimental drugs are associated with increased odds of toxic death, treatment discontinuation without disease progression and high grade AEs when compared to the standard treatment received by controls. When we evaluated individual toxicities independently, experimental agents showed increased odds for thrombocytopenia, diarrhea, stomatitis, hypertension, cardiac events, fatigue/asthenia and skin toxicity compared to the treatment in the control arms. Univariable analysis did not identify any association between PFS (the most common primary endpoint of included studies) and any of toxicity outcome measures, however there was a statistically significant association between OS and treatment discontinuation without disease progression and with skin toxicity (greater magnitude of effect) and thrombocytopenia (lower magnitude of effect). The reason for these observations is unclear and may reflect a chance finding.

The balance between benefits and risks of anti-cancer drugs extends over a spectrum of efficacy and toxicity. It is difficult to identify scenarios in which trade-offs between benefits and risks are favorable and unfavorable. Furthermore, because phase 3 trials are closely monitored and stopped at signs of futility or increased toxicity in experimental groups, the trade-off of risks and benefits for patients is very likely to be different early in the earlier phases of accrual compared to later phases.

Data suggest that industry-sponsored RCTs, which represent the majority of all RCTs^7^, are more likely to exclude elderly patients as well as those with medical comorbidities and certain concomitant medications^13^. This means that compared to real-world practice, participants of RCTs are likely to have better performance status, less comorbidity and are expected to have better tolerability of treatment. This has direct implications to routine clinical practice since drugs approved with a favorable balance between benefit and risk among participants of RCT populations may not be representative of less selected real world population in which reduced benefit and increased toxicity may be observed, limiting generalizability^5^.

A previous meta-analysis reported an increased toxicity associated with FDA approved agents^5^ with a similar magnitude of effect to that we observed in this current study exploring unselected drugs. In contrast to our study, Niraula and colleagues did not include immunotherapeutic agents. When targeted agents used as monotherapy were compared to chemotherapeutics in the analysis by Niraula et al., a lower rate of treatment discontinuation without disease progression and less hematologic toxicity were observed. However, targeted agents are more likely to be used for a prolonged time in comparison to conventional chemotherapy which is typically administered for shorter durations. This can lead to an increased risk of cumulative low grade toxicity which may not be captured in RCTs which focus more on higher grade toxicities.

In our study, no association was observed between efficacy or toxicity end points. However, individual reports for some targeted agents, support an association between improved clinical outcomes such as PFS, OS or quality of life and certain AEs (e.g. skin toxicity with EGFR inhibitors and hypertension with VEGF inhibitors)^14,15^. This can occur when inhibition of the same target is responsible for both efficacy and toxicity.

Prior data demonstrate no apparent difference in efficacy between targeted therapy where the target was an oncogene, activated oncogenic signaling pathways, angiogenesis or an immune-modulatory target^16^. However, greater improvement in PFS with drugs targeting oncogenes or activated pathways and anti-angiogenic agents as compared to immunotherapy and conventional cytotoxic drugs was seen. This finding may reflect that PFS may not be the optimal endpoint for trials evaluating immunotherapy^17^. Of note, immunotherapy was associated with a more favorable safety and toxicity outcomes compared with other forms of targeted therapy or cytotoxic chemotherapy^16^. These results should be interpreted with caution as immune-related events may be sub-optimally reported in RCTs^18^ as classification of AEs have been based on the Common Terminology Criteria for Adverse Events (CTCAE)^19^, which may underestimate some immune-related AEs^20^. In contrast, some of the individual severe AEs such as diarrhea, skin toxicity and dyspnea (as surrogates for colitis, dermatitis and pneumonitis) may be captured in the analysis^16^.

Of note, our sensitivity analysis showed that blinded studies were associated with significantly higher odds for treatment discontinuation and several grade 3/4 AEs. These findings additionally strengthen our general results and raise concern about under-reporting of toxicity in open-label trials. It has been suggested that patient-reported outcomes (PROs) are a key outcome measure of clinical trials regardless of blinding status and thus improved design could help ensure high-quality data which may inform patient-centered care^21^.

Since the majority of studies included in this analysis investigated efficacy and toxicity of targeted agents such as small molecules (most commonly kinase inhibitors) it is important to highlight key lessons learned from pivotal trials of this group of drugs. It has been suggested that such drugs should undergo testing of more than one dose in phase 2 trials, incorporating biomarker and target inhibition data when the mechanism of action is clear and continuously evaluating dosing and dosing regimens throughout drug development. The observation of treatment-related death in phase 3 trials is highly undesirable^22^.

Our analysis has limitations. First, it is based on clinical trial reports and not on individual patient data. Second, significant heterogeneity was seen between trials. In some trials the control group was an approved active treatment, whilst in others it was a placebo or best supportive care. This has an important impact on the observed relative benefit and relative toxicity. Third, we included data on only 4 common solid tumors, thus limiting generalizability to other tumor types; however, these 4 groups of tumors represent the largest cancer burden worldwide. Fourth, efficacy endpoints were reported as relative statistics and relative differences do not necessarily translate into large differences in absolute benefits^23^. Finally, the use of CTCAE more likely captures more severe acute toxicities and may not capture less severe, but chronic AEs. This makes the generalizability of AEs as a measure of overall quality of life more limited. Furthermore, an important point we would like to highlight at the end is the fact that toxicities that occur under 5% may be inadequately reported in clinical trials and thus may not be fully represented in our manuscript as we could only extracted data that were reported^24^. And last but not least, our analysis represents completed trials which are more likely to be positive so the trade-off of risk and benefits for patients we observed is somewhat different that would be if the trials were not completed.

In conclusion, only 57% of individual phase 3 RCTs in common solid tumors result in improved outcomes and many experimental drugs have worse safety and tolerability compared to control therapy. Oncologists should be aware of these risks and should disclose them to cancer patients when considering enrollment on phase 3 trials.

## Supplementary Information

Supplementary Information.

Supplementary Table 1.

Supplementary Legend.

## Data Availability

The data that support the findings of this study are available from the corresponding author upon reasonable request.

## References

[1] Harbour R, Miller J (2001). A new system for grading recommendations in evidence based guidelines. BMJ.

[2] Sackett DL, Rosenberg WM, Gray JA, Haynes RB, Richardson WS (1996). Evidence based medicine: What it is and what it isn’t. BMJ.

[3] Ioannidis JP (2009). Adverse events in randomized trials: Neglected, restricted, distorted, and silenced. Arch. Intern. Med..

[4] Pitrou I, Boutron I, Ahmad N, Ravaud P (2009). Reporting of safety results in published reports of randomized controlled trials. Arch. Intern. Med..

[5] Niraula S (2012). The price we pay for progress. A meta-analysis of harms of newly approved anticancer drugs. JCO..

[6] www.clinicaltrials.gov. Accessed Sept 2016.

[7] Ribnikar Domen (2018). Reporting of randomized trials in common cancers in the lay media. Oncology.

[8] Der Simonian R, Laird N (1986). Meta-analysis in clinical trials. Control Clin. Trials..

[9] Deeks, J. J., Higgins, J. P. T. & Altman, D. G. *Analysing and **Presenting Results: Cochrane Handbook for Systematic Reviews of Interventions 4 2 5*. (Wiley, 2006).

[10] Sweeting M, Sutton A, Lambert P (2004). What to add to nothing? Use and avoidance of continuit corrections in meta-analysis of sparse data. Stat. Med..

[11] Stanley TD, Doucouliagos H (2017). Neither fixed nor random: Weighted least squares meta-regression. Res.. Synth. Methods..

[12] Chan JK (2008). Analysis of phase II studies on targeted agents and subsequent phase III trials. What are the predictors for success?. J. Clin. Oncol..

[13] Van Spall HG, Toren A, Kiss A, Fowler RA (2007). Eligibility criteria of randomized controlled trials published in high-impact general medical journals: A systematic sampling review. JAMA.

[14] Peeters M (2009). Association of progression-free survival, overall survival, and patient-reported outcomes by skin toxicity and KRAS status in patients receiving panitumumab monotherapy. Cancer.

[15] Rini BI (2011). Hypertension as a biomarker of efficacy in patients with metastatic renal cell carcinoma treated with sunitinib. J. Natl. Cancer Inst..

[16] Barnes TA (2017). Efficacy, safety, tolerability, and price of newly approved drugs in solid tumors. Cancer Treat. Rev..

[17] Amir E, Seruga B, Kwong R, Tannock IF, Ocana A (2012). Poor correlation between progression-free survival and overall survival in modern clinical trials: Are composite endpoints the answer?. Eur. J. Cancer..

[18] Chen TW, Razak AR, Bedard PL, Siu LL, Hansen AR (2015). A systematic review of immune-related adverse event reporting in clinical trials of immune checkpoint inhibitors. Ann. Oncol..

[19] Common Terminology Criteria for adverse events v 4.0, in NCI, NIH, DHHS. May 29, 2009. N.C. Institute, Editor. NIH: NIH publication # 09-7473 (2009).

[20] Spain L, Diem S, Larkin J (2016). Management of toxicities of immune checkpoint inhibitors. Cancer Treat. Rev..

[21] Calvert M (2018). Guidelines for inclusion of patient-reported outcomes in clinical trial protocols: The SPIRIT-PRO extension. JAMA.

[22] Bullock JM, Rahman A, Liu Q (2016). Lessons learned: Dose selection of small molecule-targeted oncology drugs. Clin. Cancer Res..

[23] Amir E (2011). Oncogenic targets, magnitude of benefit, and market pricing of antineoplastic drugs. J. Clin. Oncol..

[24] Seruga B (2016). Under-reporting of harm in clinical trials. Lancet Oncol..

