# Supplementary material for: Efficacy, safety and tolerability of drugs studied in phase 3 randomized controlled trials in solid tumors over the last decade
Source: Sci Rep. 2021 May 25;11:10843. doi: 10.1038/s41598-021-90403-3 (PMC8149406; doi:10.1038/s41598-021-90403-3)
Supplement: Supplementary file 1 — Supplementary Information. [file 41598_2021_90403_MOESM1_ESM.doc]

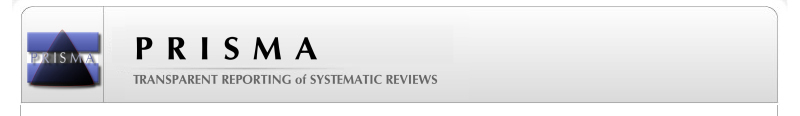
**PRISMA 2009 Flow Diagram**

**Screening**

**Included**

**Eligibility**

**Identification**

Records identified through database searching
(n = 377)

Additional records identified through other sources
(n = 0)

Records after duplicates removed
(n = 0)

Records screened
(n = 143)

Records excluded
(n =234)

42 active trials without results

37 adjuvant trials

36 trials of other cancers

32 supportive care trials

19 single arm studies

16 different scheduling and/or dosing of the same drug

13 radiation trials

10 surgical trials

10 prevention trials

9 imaging trials

3 studies of the same drug/class of drugs

2 biomarker analyses

1 pharmacokinetic/pharmacodynam-ic study

1 other than antitumoral effect

2 completed before 2005

1 follow up of the study completed in 2000

Full-text articles assessed for eligibility
(n =143)

Full-text articles excluded, with reasons
(n = 27)

Studies included in qualitative synthesis
(n = 116)

Studies included in quantitative synthesis (meta-analysis)
(n = 116)
