# Supplementary material for: Efficacy, safety and tolerability of drugs studied in phase 3 randomized controlled trials in solid tumors over the last decade
Source: Sci Rep. 2021 May 25;11:10843. doi: 10.1038/s41598-021-90403-3 (PMC8149406; doi:10.1038/s41598-021-90403-3)
Supplement: Supplementary file 3 — Supplementary Legend. [file 41598_2021_90403_MOESM3_ESM.docx]

Supplementary table legend

Column A: NCT number – the number of a clinical trial found in the Clinicaltrials.gov database

Column B: drug – a drug that was evaluated in a specific trial

Column C: control arm agent – either placebo or an active agent that was used for patients in a control arm of a specific study

Column D: class of a drug – either a targeted agent, chemotherapeutic, endocrine agent, immunotherapeutic or other

Column E: blinded/open label - a concealment method of a trial: either a study was blinded or an open label

Column F: cancer site: a site/localisation of a cancer; either breast, prostate, lung or colorectal

Column G: setting of a trial; either adjuvant or palliative; but we included only palliative trials, so all of them are classified as palliative

Column H: date of publication: a date of publication of the study results in a journal

Column I: date of reporting on a conference: a date when a study was presented on a conference

Column J: result: a result of a trial; either positive or negative

Column K: primary endpoint: a primary endpoint of a specific study; either overal survival (OS), progression-free survival (PFS), disease-free survival (DFS), response rate (RR), other

Column L: the result of a primary endpoint: either hazard ratio (HR) or relative ratio (RR)
